# A Strong Case for Viral Genetic Factors in HIV Virulence

**DOI:** 10.3390/v3030204

**Published:** 2011-03-08

**Authors:** Viktor Müller, Christophe Fraser, Joshua T. Herbeck

**Affiliations:** 1 Institute of Biology, Eötvös Loránd University, Pázmány P. s. 1/C, 1117 Budapest, Hungary; 2 Department of Infectious Disease Epidemiology, School of Public Health, Imperial College London, London W2 1PG, UK; E-Mail: c.fraser@imperial.ac.uk; 3 Department of Microbiology, University of Washington School of Medicine, Seattle, WA 98195, USA; E-Mail: herbeck@uw.edu

**Keywords:** HIV virulence, heritability, viral factors

## Abstract

HIV infections show great variation in the rate of progression to disease, and the role of viral genetic factors in this variation had remained poorly characterized until recently. Now a series of four studies [1–4] published within a year has filled this important gap and has demonstrated a robust effect of the viral genotype on HIV virulence.

Infection with Human Immunodeficiency Virus Type 1 (HIV-1) is nearly always fatal without treatment; however, the time from infection to death varies from less than one year to more than two decades [5,6]. Uncovering the factors behind this variation in HIV virulence (rate of disease progression) might provide important clues for the understanding and management of the disease. There are three main sources of the variation: host genetic factors, viral genetic factors and environmental variability—with potential interactions between the categories. In recent years, great effort has been put into elucidating and quantifying the role of host genetic factors [7–12]; however, systematic studies on the contribution of viral genetic factors had remained scarce. Hints for the role of viral factors included differences in virulence based on viral subtype [13–15], co-receptor use [16–19], or the presence of deleterious mutations in the virus [20,21]. However, the extent of heritable variation in HIV virulence, and its relevance in the epidemic, can only be assessed quantitatively by inspecting transmission pairs or networks. Two early reports on transfusion-associated infections demonstrated that the rate of disease progression between donors and recipients was correlated within transmission pairs [22,23]. However, transmission pairs are rarely identified, and the next study of this kind followed after a gap of 10 years [24], and was already from the HAART (Highly Active Antiretroviral Therapy) era. Because effective antiretroviral therapy is able to halt or slow disease progression, HIV virulence now needs to be characterized by proxies, such as plasma virus load, that are correlated with the rate of progression in untreated infections [25]. The study of Tang *et al.* found a significant correlation of log10 HIV-1 RNA levels between transmitting (source) and seroconverting (recipient) heterosexual partners in Zambia [24], which was consistent with the earlier findings of the pre-HAART era. Then last year witnessed a breakthrough with the publication of four new studies [1–4], which all reported the correlation of virus load in phylogenetically linked transmission pairs, thus robustly corroborating the effect of viral genetic factors on HIV virulence. Below we describe the range of methods and study populations investigated in these studies (Table 1), and then discuss the interpretation and implications of the results. For completeness, we include also the earlier study of Tang *et al.* [24] in our discussion.

Four of the five studies investigated the correlation of virus loads within transmission pairs [1,2,4,24]. Such pairs were identified either as HIV-1 serodiscordant couples with subsequent HIV transmission [1,24] or by contact tracing of individuals with recent acute infection [2,4]; close relatedness of viruses between source and recipient was validated by phylogenetic analyses in all studies. The “heritability” of virus load, or couple effect, was assessed by Pearson correlation coefficient, *r*, between the virus loads of source and recipient individuals [2,4,24], or by general linear models controlling for potential confounders [2,24]. Hecht *et al.* reported that the couple effect remained significant when controlling for age, ancestry and human leukocyte antigen (HLA) class I alleles, and none of the co-factors had significant effect [2]; Tang *et al.* also estimated very similar coefficients with Pearson correlation and with general linear models controlling for age, gender and HLA class I markers [24]. In contrast, Hollingsworth *et al.* used analysis of variance (ANOVA) to estimate the proportion of variance in the virus loads that could be explained by similarity within transmission pairs [1]. Finally, Alizon *et al.* applied the phylogenetic comparative approach (PCA [26]) to estimate a “phylogenetic signal”, which correlates similarity in the virus loads with similarity (relatedness) between viral sequences within an epidemiologically linked cluster of individuals [3]. This approach does not require knowledge of transmission pairs, but works best with clusters of individuals who are not far removed in the transmission network.

In spite of the differences in methodology, the estimates from all studies can be brought to a common ground. The relevant quantity to be estimated is the proportion of variance in the virus load that is explained by viral genetic factors (*i.e.*, heritability [27]). Assuming that the similarity within transmitting pairs arises from the similarity of the viral genotypes, the coefficient of determination from ANOVA provides a robust estimate of this proportion [1]. It can also be shown that the Pearson correlation coefficient equals the coefficient of determination, provided the virus loads of source and recipient individuals are normally distributed with identical variance [1,28]; this equivalence was validated numerically on the data analyzed in [1]. Finally, the phylogenetic signal correlates very strongly with heritability, with nearly identical expected values at low to moderate heritability [3]. Thus, the coefficients derived by all three methods (and all five papers) provide numerically comparable estimates of the heritability of HIV virulence.

The finding of considerable heritability in all five studies that encompassed five different countries across three continents, with patient cohorts characterized by European [2–4] or African [1,24] ancestry, subtype B [2–4], C [24] or A and D [1], and heterosexual [1,24] or same-sex [2,3] exposure, argues strongly for a robust effect of viral genetic factors in HIV virulence. Why then are the estimates of heritability so different among the studies? The selected main results of the studies (Table 1) show three-fold variation in the magnitude of heritability, and there is even greater variation if the results for alternative patient subsets or virus load definitions are considered. A great part of this variation might simply reflect the random deviation of the estimates: all studies involved relatively small samples, and due to the heterogeneity of the datasets, some estimates were based on subsamples. Of note, the 95% confidence interval of the estimate by Hecht *et al.* (0.19–0.78) [2] includes the point estimates of the other four studies. However, some of the variation might reflect systematic differences or confounding factors. Below, we discuss some possible sources of such variation.

## Calculation of Virus Load in the Recipient

First, the studies differed in the definition or calculation of the virus load in the recipients. Three studies [1,3,24] estimated “set point” (*i.e.*, chronic) virus load that is attained after the resolution of primary infection, and has been shown to correlate with subsequent disease progression [25]. In contrast, two studies [2,4] used the magnitude of the acute peak of virus load in the recipient patient, which is also a prognostic marker for the subsequent course of infection [29]; however, even these studies likely had measures of chronic virus load from most of the source individuals. Because different viral factors might act (and with different relative contribution) during acute and chronic infection, one could expect a stronger correlation between chronic-chronic than between chronic-acute virus loads. This expectation was borne out in the study of Hecht *et al.*, where the correlation was stronger when chronic virus loads were considered (*r* = 0.71 *vs.* 0.55 in the chronic-acute analysis) [2]. In contrast, van der Kuyl *et al.* found a stronger correlation (*r* = 0.29) when recipients were in early acute infection (Fiebig stages III or IV, generally less than 30 days after viral transmission [30]), compared when recipient virus loads were assayed in later stages of infection (Fiebig stages V, greater than 30 days after transmission, or VI, greater than 100 days after transmission) (*r* = 0.06) [4]. However, none of the datasets analyzed were sufficiently large to address this issue with any degree of confidence.

## Calculation of Virus Load in the Source

Second, the definition of set point or chronic virus load in the source individuals might also be relevant. Hollingsworth *et al.* [1] and Alizon *et al.* [3] used the mean of several measurements (where available); in addition, the latter included only individuals in whom fluctuations of the virus load remained within a 10-fold range. Tang *et al.* used a single chronic data point from each individual [24]. Finally, Hecht *et al.* [2] and van der Kuyl *et al.* [4] used a single virus load from the source (probably also in chronic stage in most cases), matched in time as closely as possible to the acute data point of the recipient. The definition of the chronic virus load might affect heritability estimates depending on the nature of the within-host evolution of HIV. Given the great evolutionary capacity of the virus, if genetic factors affect virulence, these might also change during the course of an infection [31–33]. If these changes are primarily host-specific, then adaptation to one host might be neutral or even maladaptive in another. In this case, the apparent heritability of virulence would decrease with the age of infection in the source, even though the effect of viral genetic factors does not. For example, immune escape mutations might generate increasing virulence in an individual; however, these mutations might be disadvantageous for the virus in another host with different MHC alleles [34–36], and therefore the host-specific component of increased virulence would be annulled or even reversed in recipients. In contrast, if the within-host evolution of virulence factors is primarily not host-specific, then heritability should be highest when the virus loads of source and recipient are sampled as close in time as possible, minimizing the evolutionary divergence between the two virus populations. Both Hecht *et al.* and van der Kuyl *et al.* used virus loads close to the transmission event; however, one of these studies [2] found relatively strong, the other [4] relatively weak heritability: it is therefore not possible to infer the nature of within-host evolution from these results. Finally, if within-host evolution has negligible effect on viral virulence factors, then the viral component of virulence stays constant during the course of an infection, and the time of sampling should not matter. In this case, highest heritability would be expected when the mean of several data points is used (as in [1,3]) or when several measurements are considered in a repeated measures analysis [27]. These approaches reduce, or control for, the effect of random variation on the measurements, which would result in a stronger relative effect of viral genetic variation and hence greater heritability. The nature of within-host virulence evolution (host-specific, generic, negligible) could thus be gleaned by comparing heritability estimates calculated using different definitions of the virus load (earliest, closest to transmission or mean/repeated measures) in the same patient population.

## Non-Viral Factors

Third, several non-viral confounding factors might bias the heritability estimates towards both under- and overestimation. Shared environmental factors within transmission pairs (or among closely related cases in [3]) might result in the overestimation of heritability. Any non-viral factor that correlates within transmission pairs increases the “couple effect”, which has been interpreted as heritability. In contrast, imperfect identification of transmission pairs results in underestimation, because the viruses in some of the pairs might then not be closely related. Indeed, Hollingsworth *et al.* found stronger heritability when only pairs with the highest phylogenetic support for transmission were included in the analysis [1]. The PCA method used by Alizon *et al.* was shown to be robust to errors in the phylogenetic reconstruction [3]. However, the method found strong heritability only when restricted to the densely sampled and connected MSM (men having sex with men) transmission group, but not when including all transmission groups. This indicates that general sequence similarity (relatedness) is not a good predictor of virulence factors when two virus strains are not closely related. Such a pattern can be expected if either virulence factors and/or the genomic region used for the phylogenetic reconstruction evolve quickly and under different selection pressures.

Another confounding effect might arise from the influence of virus load on transmission, as has been pointed out by Tang *et al.* [24]. Higher virus load correlates with a higher probability of transmission, which implies that transmitters tend to have virus loads above the average of the population or that of their seroconverter partners—which was indeed found in both studies that included chronic data points from the recipient partners of transmission pairs [1,24]. This bias skews the distribution of non-viral factors towards the regimes supporting high virus load in the transmitters, and thereby reduces the relative contribution of viral genetic factors to virulence—and thus also heritability.

In principle, the “dose” of transmitted virus could also influence the outcome of an infection. Higher virus load in the source individual might be associated with a higher transmitted dose. If a higher dose resulted in higher set point in the recipient, this could generate correlated set points within transmission pairs independent of viral genetic factors. Unfortunately, the study of heritability in transmission pairs cannot easily distinguish between genuine (genetic) heritability and dose effect. However, while dose certainly has an effect on the probability of successful transmission, its potential influence on the chronic virus load is much less clear. Most mathematical models of HIV dynamics predict that the virus load has a single “stable” steady state at any given point in the course of the disease, determined by viral and host parameters [37,38]. This steady state is attained irrespective of the starting conditions, e.g., variation in the infectious dose. The stability of the steady state (set point) is confirmed by its resilience to perturbations, e.g., virus load tends to return to pre-treatment levels after the cessation of effective antiretroviral therapy [39]. However, the role of the infectious dose cannot be excluded completely. Some mathematical models allow for the establishment of efficient virus control (and an alternative low set point) if the HIV-specific CD4+ T cell response is preserved at the beginning of an infection [40,41]—which could, in principle, happen at a low infectious dose (and might be facilitated by antiretroviral treatment during acute infection [42]). Furthermore, the transmitted dose might affect the peak virus load in acute infection, even if the subsequent set point is not affected. This could, at least partially, explain the chronic-acute virus load correlations that were found in some of the studies discussed [2,4].

However, while there is so far no empirical evidence in support of a dose effect in HIV infection, some observations argue indirectly against it. It has been shown recently that most infections are initiated by a very low effective dose (a single viral clone in 60–80% of the cases [43,44]). Although there is some variation in the number of initially transmitted viral clones (1–10 clones were estimated in [44]), this variation is comparable in magnitude to the amplification of the viral stock in a single round of the viral life cycle (estimates of the basic reproductive ratio in acute HIV infection range between 3–34 [45,46]). Even the highest dose could thus give only a few days head start for the infection. Furthermore, in contrast to the estimations based on transmission pairs, the phylogenetic signal estimated by Alizon *et al.* on a loosely associated transmission network [3] is likely to have been insensitive to a potential dose effect. We therefore conclude that most of the available evidence argues against the relevance of a dose effect in the estimated heritability of the virus load.

## Inherent Variability of Heritability

Finally, we must emphasize that heritability is an inherently variable measure, rather than a well-defined characteristic of a trait within a species. Heritability expresses the relative contribution of genotypic to phenotypic variability. This relative contribution depends on the variability of both the genotype and of the environmental factors (in this sense including also the host genetic factors) that affect phenotype, and is further confounded by the interactions between the two [27]. Because of this, heritability characterizes a given population (with a given genotypic variance) in a given environment (with a given variance), and can vary considerably across populations of the same species. For example, more variability in the virus population (e.g., Central Africa *vs.* Europe) allows for stronger heritability of viral traits (including virulence); in contrast, greater genetic variability in the host population could reduce their heritability (e.g., greater human variability in Africa *vs.* Europe could reduce the heritability of HIV virulence). Shared environmental factors within a transmission group (e.g., potentially among MSM) would reduce environmental variance and thereby increase heritability. In this light, it might be relevant that the two studies restricted to MSM in high-income countries [2,3] found stronger heritability than those that included several risk groups or were conducted in low-income countries with probably greater environmental variability. In contrast, heterogeneity in the virus subtype might increase heritability; however, the only study that included several subtypes did control for their potential differences [1].

Partly due to the inherent complexity of heritability, it is difficult to make a meaningful comparison with the estimated effect of host factors [7–12] (host factors identified so far could explain about 20% of the variation in the rate of disease progression [7]). Furthermore, the study of viral factors in transmission pairs who are infected with closely related or identical viruses would be analogous to the study of host factors in identical twins infected with different virus variants—which is clearly impossible. The effect of host factors is instead investigated with a high-resolution mapping of genomic variation in the infected individuals; however, this approach cannot detect variants that are rare or that have only a weak effect on disease progression. In addition, the effect of host and viral factors might not be independent, e.g., the effect of an MHC allele depends on the presence of the epitopes that it can present within the viral genome. These factors imply that the relative contribution of host and viral factors to the viral set point might vary and cannot be compared on the basis of the estimates for heritability and host effects.

Considering all potential confounding effects and the inherently variable (population-specific) nature of heritability, the variation in the coefficients estimated across several continents, in different populations and with different methods, should not come as a surprise. In fact, the consistent finding of a heritable component of virus loads across these studies argues convincingly for a strong effect of viral genetic factors on HIV virulence—which has important implications.

## Implications

Heritable variation, if it affects survival or reproduction, sets the stage for the adaptive evolution of a trait. For HIV, we see that plasma virus load varies among individuals, is associated with transmission, and is heritable; thus the conditions for adaptive evolution at the population (between host) level are in place. Based on the dependence of both transmission probability and (untreated) survival time on the virus load, it has been possible to estimate the virus load that maximizes the transmission potential of HIV [47] in the absence of treatment. The estimate is close to the current mean virus load in the epidemic, implying that the virulence of HIV might already have approached its optimum, and might not be likely to evolve toward attenuation [47]. Indeed, recent analyses of time trends in clinical markers of virulence in large patient cohorts have found mostly stable [48–51] or increasing [52–55] virulence. A meta-analysis of time trend studies found that virulence has been increasing at a slowing rate over the last three decades, which is consistent with evolution toward an optimum [56].

However, the evolution of HIV might be dominated by the selection forces acting at the within-host level [57], in which case selection for the efficiency of between-host transmission can take only weak effect [32,58]. Traits affecting virulence might nevertheless be under selection also at the within-host level [58]. If virulence were linked directly to replication efficiency, then HIV would be expected to evolve toward the level of virulence associated with the rate of replication that is optimal for the virus within the host [59]. In contrast, if virulence is primarily a “side effect”, arising from a mechanism (e.g., generalized immune activation [60–62]) not directly linked to virus replication, then within-host selection might not drive the evolution of virulence [63].

The heritability and, therefore, evolvability of HIV virulence might also have implications for the optimal treatment strategies at both the individual and the population level. Human interventions can potentially affect the selection forces acting on virulence (e.g., by altering the effect of viral virulence factors on transmission and survival), and could therefore provoke an evolutionary response from the virus, with potentially harmful or beneficial consequences.

## Possible Mechanisms

Finally, a crucial next step will be to elucidate the viral factors that affect virulence. Such factors might fall into three broad categories. First, genomic variation might affect the replication capacity of the virus, which influences steady-state virus load through the production rate of virus particles. The existence of such factors is evidenced by attenuated virus clades containing deleterious mutations in nef [20] or in other accessory genes [21], and by the “fitness costs” associated with drug resistance [64–66] or immune escape [35,67] mutations in the absence of drug or immune pressure. Second, genomic variation can also affect the ability of the virus to evade immune responses, which affects the steady-state virus load through the rate of clearance. Adaptation of HIV to the human immune response can affect virulence both within a host (reviewed in [68]) and, in the case of frequent host genotypes, also at the population level [69,70]. Finally, in addition to virus load, some viral factors might also affect the “per capita” pathogenicity of HIV, *i.e.*, the “conversion rate” between virus load and virulence. If much of the pathogenicity arises as a side effect (e.g., through chronic immune activation [60–62]), then the ability of the virus to induce this side effect might also vary [63].

A better understanding of these factors could provide important clues for the development of novel treatment strategies that could be aimed at mitigating the pathogenic effect of the virus. With the existence of viral virulence factors firmly established, the search for the individual factors can continue.

## Figures and Tables

**Table 1.** Studies that estimated the heritability of HIV virus load.

**Study**	**n**	**Route of transmission**	**Country**	**VL compared**	**Method**	**Estimated heritability [95% CI]**
Tang et al. [24]^a^	115^b^	HET	Z	chronic	LC	0.20^c^ [0.01–0.37]^d^
Hollingsworth *et al.* [1]	97^b^	HET	UG	chronic	ANOVA	0.23^e^ [n.a.]
Hecht et al. [2]	24^b^	MSM	US	acute *vs.* chronic^f^	LC	0.55^c^ [0.19–0.78]
Alizon *et al.* [3]	134^g^	MSM	CH	chronic	PCA	0.59^h^ [0.45–0.73]
van der Kuyl *et al.* [4]	56^b^	HET, MSM	NL	acute *vs.* chronic^f^	LC	0.25c [−0.01–0.48]^d^

Abbreviations: VL—virus load; HET—heterosexual; MSM—men having sex with men; LC: linear correlation; ANOVA: analysis of variance; PCA—phylogenetic comparative approach; n.a.: not available. Countries: Z—Zambia, US—United States, UG—Uganda, NL—the Netherlands, CH—Switzerland.

a This earlier study was included for completeness.

b Number of transmission pairs.

c Correlation coefficient.

d Calculated with Fisher Transformation using the point estimate and sample size.

e Coefficient of determination.

f Acute virus load in the secondary case was correlated with chronic virus load in the index patient.

g Number of patients.

h Phylogenetic signal [26].
